# Self-compassion predicts reduced bedtime procrastination through affect pathway: a three-wave longitudinal study

**DOI:** 10.1186/s40359-026-05267-x

**Published:** 2026-07-31

**Authors:** Fangbai Dong, Siqi Xie, Ziyi Yan, Yi Feng, Yaming Hang

**Affiliations:** 1 Mental Health Education Center, Henan Agricultural University, Zhengzhou, China; 2 Mental Health Education Center, Shanghai Songjiang No.2 High School, Shanghai, China; 3 Faculty of Psychology, Beijing Normal University, Beijing, China; 4 Mental Health Center, Central University of Finance and Economics, No.39 South College Road, Haidian District, Beijing, 100081 China; 5 School of Sociology and Psychology, Central University of Finance and Economics, Beijing, China; 6 Fujian Province Key Laboratory of Applied Cognition and Personality, No. 36 Xianqianzhi Street, Xiangcheng District, Zhangzhou, 363000 China; 7 Institute of Applied Psychology, Minnan Normal University, Zhangzhou, China; 8 School of Education and Psychology, Minnan Normal University, Zhangzhou, China

**Keywords:** Bedtime procrastination, Self-compassion, Positive affect, Negative affect, Longitudinal study

## Abstract

**Background:**

Bedtime procrastination is a prevalent self-regulation failure phenomenon among college students, linked to poor sleep quality and mental illness. There are many factors contributing to bedtime procrastination. Among them, self-compassion may alleviate it through its influence on emotional states, yet few studies have researched in-depth on this mechanism.

**Methods:**

We conducted a three-wave longitudinal study with 7,174 Chinese college students (*M*_age_ = 19.11; 62.8% female) over an 8-month period. Self-compassion, positive affect, negative affect, and bedtime procrastination were measured at each wave. Cross-lagged panel modeling was used to examine temporal associations and indirect effects of self-compassion on bedtime procrastination through affective states.

**Results:**

Significant correlations among self-compassion, positive affect, negative affect, and bedtime procrastination at different waves were observed (all *p* < 0.001). Self-compassion predicted reduced bedtime procrastination over time both directly (*β* = −0.051, *p* < 0.001) and indirectly via increased positive affect (*β* = −0.007, *p* < 0.001) and decreased negative affect (*β* = −0.005, *p* < 0.001).

**Conclusions:**

This study provides the first longitudinal evidence that both positive and negative affect mediate the association between self-compassion and bedtime procrastination. These findings support emotion regulation as a key mechanism linking self-compassion to adaptive sleep behavior. Enhancing self-compassion may be a promising target for interventions aimed at reducing bedtime procrastination and improving sleep health among university students.

**Supplementary Information:**

The online version contains supplementary material available at https://doi.org/10.1186/s40359-026-05267-x.

## Background

Bedtime procrastination, defined as the voluntary and unnecessary delay of going to bed without external reasons [1], is a widespread and growing concern, particularly among young adults and college students [2]. Despite knowing the adverse consequences of sleep deprivation, individuals often postpone sleep to engage in leisure activities such as browsing social media, watching videos, or playing games [3]. This behavior has been found to be associated with shorter sleep duration, lower sleep quality, and increased daytime fatigue [4]. As a form of maladaptive self-regulation, bedtime procrastination undermines sleep, which is a fundamental component of both mental and physical health [5], and has therefore received increasing research attention in recent years.

One emerging line of inquiry investigates the psychological factors that protect against bedtime procrastination. Among them, self-compassion, defined as a kind, mindful, and nonjudgmental attitude toward oneself during times of struggle [6], has shown promise. Individuals with higher self-compassion are more likely to practice health-promoting behaviors and less likely to engage in avoidance-based or harmful behaviors [7, 8]. Indeed, emerging cross-sectional studies suggest that higher self-compassion is associated with less engagement in bedtime procrastination [4], though the mechanisms underlying this relationship remain insufficiently understood.

One possible explanation for this link lies in the role of emotion-related self-regulatory processes embedded within self-compassion. Rather than being equivalent to a general emotion regulation construct, self-compassion can be conceptualized as a self-regulatory emotional resource system that shapes how individuals respond to stress, failure, and daily goal violations. Individuals with higher self-compassion tend to experience more adaptive emotional functioning, characterized by higher positive affect and lower negative affect [9, 10]. In addition, they are more likely to engage in adaptive emotion regulation strategies, such as cognitive reappraisal and acceptance, and less likely to rely on maladaptive strategies such as self-blame and rumination [11–13]. These patterns suggest that self-compassion may serve as an important emotional buffer, helping individuals to maintain a balanced affective state even when faced with daily stress and unmet goals.

Meanwhile, affective states themselves are closely linked to bedtime procrastination. Individuals often delay sleep either to prolong pleasant emotions associated with enjoyable activities (i.e., reward-driven procrastination) or to avoid unpleasant emotions and anxiety experienced before bedtime (i.e., avoidance-driven procrastination) [14]. These insights highlight the importance of both positive and negative affect in shaping bedtime procrastination tendencies, and point to emotion regulation as a key leverage point for intervention. Negative affect in particular has been consistently associated with greater bedtime procrastination, possibly because individuals seek short-term emotional relief at the expense of long-term health goals [15, 16].

To better understand how self-compassion influences bedtime procrastination, the Temporal-Affective Self-Regulation Resource (TASRR) model offers an integrative framework [17]. This model posits that self-regulatory behavior is supported by internal emotional resources (i.e., higher positive affect and lower negative affect) that enable individuals to maintain health-related goals [13, 18, 19]. From this perspective, self-compassion—comprising self-kindness, common humanity, and mindfulness [20]—promotes adaptive emotional responding when individuals face difficulties in goal pursuit. Specifically, self-compassion fosters higher positive affect and lower negative affect by encouraging acceptance, cognitive reappraisal, and reduced self-criticism, thereby functioning as an internal affective resource for self-regulation [11, 21]. Empirical studies consistently show that self-compassion is associated with more adaptive emotion regulation strategies and healthier affective profiles [22]. Consistent with the TASRR model, positive affect can serve as a self-regulatory resource that supports sustained health behaviors, whereas negative affect may impair self-regulation by increasing the likelihood of short-term emotion-driven decisions. Accordingly, individuals high in self-compassion are expected to possess more effective affective resources that support self-control and reduce engagement in maladaptive behaviors such as bedtime procrastination. However, empirical research testing this full mediational pathway has been limited. Prior studies have primarily relied on cross-sectional data and have typically found only negative affect, but not positive affect, to mediate the self-compassion-bedtime procrastination link [16, 23], leaving unanswered questions about the temporal dynamics and dual affective pathways involved.

To address these gaps, the present study used a three-wave longitudinal design to examine whether both positive and negative affect mediate the relationship between self-compassion and bedtime procrastination over time. By leveraging a large sample and cross-lagged panel modeling, we aimed to test the directionality and robustness of these relationships. In line with the TASRR model, we hypothesized that self-compassion would predict lower bedtime procrastination over time, and that this relationship would be mediated by both enhanced positive affect and reduced negative affect. This study is among the first to simultaneously examine these mechanisms longitudinally, offering a more comprehensive understanding of the emotional processes through which self-compassion may protect against maladaptive sleep behaviors.

## Methods

### Participants and procedure

The present research employed a three-wave longitudinal design with a 4-month interval between adjacent waves. Data were collected via cluster sampling from a university located in north of China, where all undergraduate students across various disciplines were invited to complete an online survey via a questionnaire quick response code that was distributed by some college teachers. All participants remained anonymous, and participants were informed that they could withdraw from the survey at any time before submitting their responses. Participants also provided informed consent before completing the survey. The 4-month interval between waves was determined based on the academic calendar of university students in China. Given that bedtime procrastination is sensitive to contextual factors such as academic workload and daily routines [24], all three waves were scheduled during active semesters to minimize potential confounding effects associated with examination periods and academic vacations. This timing ensured greater contextual consistency across measurement occasions while still allowing sufficient time to capture longitudinal changes in the studied variables. Wave 1 of data collection was conducted in February 2024, and 12,233 participants (*M*_age_ = 19.54, *SD*_age_ = 1.43; 56.7% female) completed the Wave 1 survey. Approximately 4 months after Wave 1, 9054 participants (*M*_age_ = 19.32, *SD*_age_ = 1.44; 59.9% female) completed the Wave 2 survey (74.0% retention rate), and approximately 4 months after Wave 2, 8167 participants (*M*_age_ = 19.20, *SD*_age_ = 1.40; 61.6% female) completed the Wave 3 survey (66.8% retention rate). To ensure the quality of data, we applied the following exclusion criteria: (a) Participants whose answer was incorrect on the attention check question. (b) Participants who spent less than 1s on each item on average. Based on these criteria, the final samples were 11,139 for Wave 1 (*M*_age_ = 19.13 years, *SD*_age_ = 1.31; 62.2% female), and a total of 7174 participants (*M*_age_ = 19.11, *SD*_age_ = 1.30; 62.8% female) completed all three waves. To examine the pattern of missing data, Little’s Missing Completely at Random (MCAR) test was conducted. The results were significant, *χ²* [16] = 56.21, *p* < 0.001, suggesting that the missing data did not fully satisfy the assumption of MCAR. To further assess potential attrition bias, we conducted independent-samples t-tests comparing baseline levels of the key study variables between participants who completed all three waves and those who dropped out during the follow-up period. The results revealed no significant differences between the two groups on any study variable at Wave 1 (see Table S1 in the Supplementary Materials). These findings suggest that although the missingness was not strictly MCAR, participant attrition was unlikely to be systematically related to the focal study variables and therefore was unlikely to introduce substantial bias into the study findings. Of these 7174 participants, 73.2% came from rural areas and 26.8% came from urban areas. Regarding mental health history, 8.0% reported a prior diagnosis of a psychiatric disorder, and 0.4% indicated current use of psychotropic medication. This study received approval from the Research Ethics Review Committee of Beijing Normal University.

### Measures

#### Social-demographic variables

Demographic information including gender, age, education level, place of residence (i.e., city and country), mental disorder history, and socioeconomic status were collected.

#### Self-compassion

Self-compassion was measured using the Self-Compassion Scale-Short Form (SCS-SF), a validated and efficient alternative to the original long form [25]. The SCS-SF maintains the same factor structure as the full scale, demonstrates good internal consistency, and exhibits a near-perfect correlation with the original version. It has been widely adopted in international research and is applicable across diverse populations, including college students [26]. This self-report scale has been validated in Chinese people [27]. The scale comprises 12 items rated on a five-point Likert scale ranging from 1 (almost never) to 5 (almost always). Total scores range from 12 to 60, with higher scores indicating greater self-compassion. In the present study, the internal consistency of the SCS-SF was good across three waves (Wave 1: Cronbach’s α = 0.83; Wave 2: α = 0.85; Wave 3: α = 0.85). The CFA results showed that the 1-factor structure of this scale was supported (χ^2^/df = 31.361, RMSEA = 0.052, CFI = 0.988, TLI = 0.975, SRMR = 0.017).

#### Positive and negative affect

The Positive and Negative Affect Schedule (PANAS) consists of 20 items that describe various affective states (e.g., happy, upset), with 10 items each assessing positive and negative affect [28]. Respondents rated the extent to which they were currently experiencing each affective state on a five-point Likert scale ranging from 1 (very slightly or not at all) to 5 (extremely). The PANAS has demonstrated solid psychometric properties, including good discriminant validity relative to measures of anxiety and depression, as well as high internal consistency [29]. In the present study, the Positive Affect subscale (Wave 1: α = 0.91; Wave 2: α = 0.92; Wave 3: α = 0.92) and the Negative Affect subscale (Wave 1: α = 0.87; Wave 2: α = 0.90; Wave 3: α = 0.89) of the PANAS both demonstrated good internal reliability across the three waves of data collection. The CFA results showed that the 2-factor structure of this scale was supported (χ^2^/df = 40.274, RMSEA = 0.059, CFI = 0.956, TLI = 0.948, SRMR = 0.052).

#### Bedtime procrastination

Bedtime procrastination was assessed using the nine-item Bedtime Procrastination Scale (BPS), which measures individuals’ tendency to unnecessarily delay going to bed [1]. The BPS includes four positively worded items (e.g., “I go to bed later than I intended”) and five negatively worded items (e.g., “I can easily stop my activities when it is time to go to bed”), each rated on a five-point Likert scale ranging from almost never to almost always. After reverse-coding the negatively worded items, the total score is calculated, with higher scores indicating greater bedtime procrastination. This scale that has been validated in China [30]. In the present study, the internal consistency of the BPS was good across three waves (Wave 1: α = 0.89; Wave 2: α = 0.90; Wave 3: α = 0.89). The CFA results showed that the 1-factor structure of this scale was supported (χ^2^/df = 41.120, RMSEA = 0.060, CFI = 0.985, TLI = 0.971, SRMR = 0.023).

### Analytic approach

All analyses were conducted using SPSS version 23.0 and Mplus version 8.3. Preliminary analyses included descriptive statistics for the study variables and intercorrelations among all variables included in the mediation model. A cross-lagged panel model (CLPM) was tested to examine the longitudinal relationships among variables, following the approach outlined by a previous study [31]. In this model, self-compassion at each wave was specified as a predictor of positive and negative affect at the subsequent wave, which in turn were modeled as predictors of bedtime procrastination at the following wave. All variables within the same wave were allowed to correlate. Model fit was evaluated using standard indices, including the Comparative Fit Index (CFI), Tucker-Lewis Index (TLI), Root Mean Square Error of Approximation (RMSEA), and Standardized Root Mean Square Residual (SRMR). Model fit was considered acceptable when RMSEA and SRMR were ≤ 0.08, and CFI and TLI were ≥ 0.90 [32, 33]. Modification indices were examined, and additional paths were added if necessary to improve model fit. Indirect effects of Wave 1 self-compassion on Wave 3 bedtime procrastination via Wave 2 positive and negative affect were also tested. These effects were computed as the product of the path coefficients from Wave 1 self-compassion to Wave 2 affect and from Wave 2 affect to Wave 3 bedtime procrastination. Missing data and potential non-normality were addressed using maximum likelihood estimation with robust standard errors. Standardized estimates are reported in the results.

## Results

### Descriptive characteristics and correlation analyses

Descriptive statistics for the main study variables across three waves are reported in Table 1. All variables including self-compassion, positive affect, negative affect, and bedtime procrastination were measured at each wave in the longitudinal sample (*N* = 7,174). Means and Standard Deviations remained relatively stable across time.

**Table 1:** Descriptive statistics of the study variables for the longitudinal sample (*N* = 7174)

	M	SD
Variables at wave 1		
Self compassion	44.67	7.24
Positive Affect	32.37	7.32
Negtive Affect	19.77	6.05
Bedtime Procrastination	26.21	7.51
Variables at wave 2		
Self compassion	45.45	7.24
Positive Affect	32.77	7.31
Negtive Affect	19.18	6.00
Bedtime Procrastination	26.58	7.77
Variables at wave 3		
Self compassion	45.69	7.45
Positive Affect	32.73	7.37
Negtive Affect	19.46	6.29
Bedtime Procrastination	25.64	7.38

Correlational analyses (see Table 2) indicated that all variables showed significant cross-wave correlations with their corresponding values at subsequent time points (*p* < 0.001), suggesting good temporal stability. Within each wave, self-compassion was positively associated with positive affect and negatively associated with negative affect and bedtime procrastination (*p* < 0.001). Positive affect was negatively correlated with both negative affect and bedtime procrastination (*p* < 0.001), whereas negative affect was positively correlated with bedtime procrastination (*p* < 0.001). Moreover, self-compassion at wave 1 was positively correlated with positive affect at waves 2 and 3, and negatively correlated with both negative affect and bedtime procrastination at later waves (*p* < 0.001). Similar cross-lagged patterns were observed for the other variables, indicating consistent directional associations across time.

**Table 2:** Correlations between model variables, at waves 1, 2, and 3 (*N* = 7174)

	2	3	4	5	6	7	8	9	10	11	12
1.Self compassion wave 1	0.549^***^	− 0.497^***^	− 0.466^***^	0.510^***^	0.376^***^	− 0.322^***^	− 0.331^***^	0.460^***^	0.349^***^	− 0.293^***^	− 0.315^***^
2.Positive Affect wave 1		− 0.280^***^	− 0.411^***^	0.354^***^	0.507^***^	− 0.178^***^	− 0.264^***^	0.336^***^	0.464^***^	− 0.184^***^	− 0.264^***^
3.Negtive Affect wave 1			0.399^***^	− 0.315^***^	− 0.192^***^	0.431^***^	0.256^***^	− 0.282^***^	− 0.191^***^	0.371^***^	0.224^***^
4.Bedtime Procrastination wave 1				− 0.335^***^	− 0.302^***^	0.275^***^	0.523^***^	− 0.299^***^	− 0.283^***^	0.258^***^	0.467^***^
5.Self compassion wave 2					0.573^***^	− 0.525^***^	− 0.489^***^	0.599^***^	0.444^***^	− 0.391^***^	− 0.399^***^
6.Positive Affect wave 2						− 0.279^***^	− 0.452^***^	0.438_***_	0.594^***^	− 0.262^***^	− 0.262^***^
7.Negtive Affect wave 2							0.415^***^	− 0.386^***^	− 0.260^***^	0.498^***^	0.317^***^
8.Bedtime Procrastination wave 2								− 0.392^***^	− 0.376^***^	0.332^***^	0.621^***^
9.Self compassion wave 3									0.593^***^	− 0.527^***^	− 0.500^***^
10.Positive Affect wave 3										− 0.287^***^	− 0.482^***^
11.Negtive Affect wave 3											0.428^***^
12.Bedtime Procrastination wave 3											/

^***^ *p* < 0. 001

### Mediation analysis

**Fig. 1:**
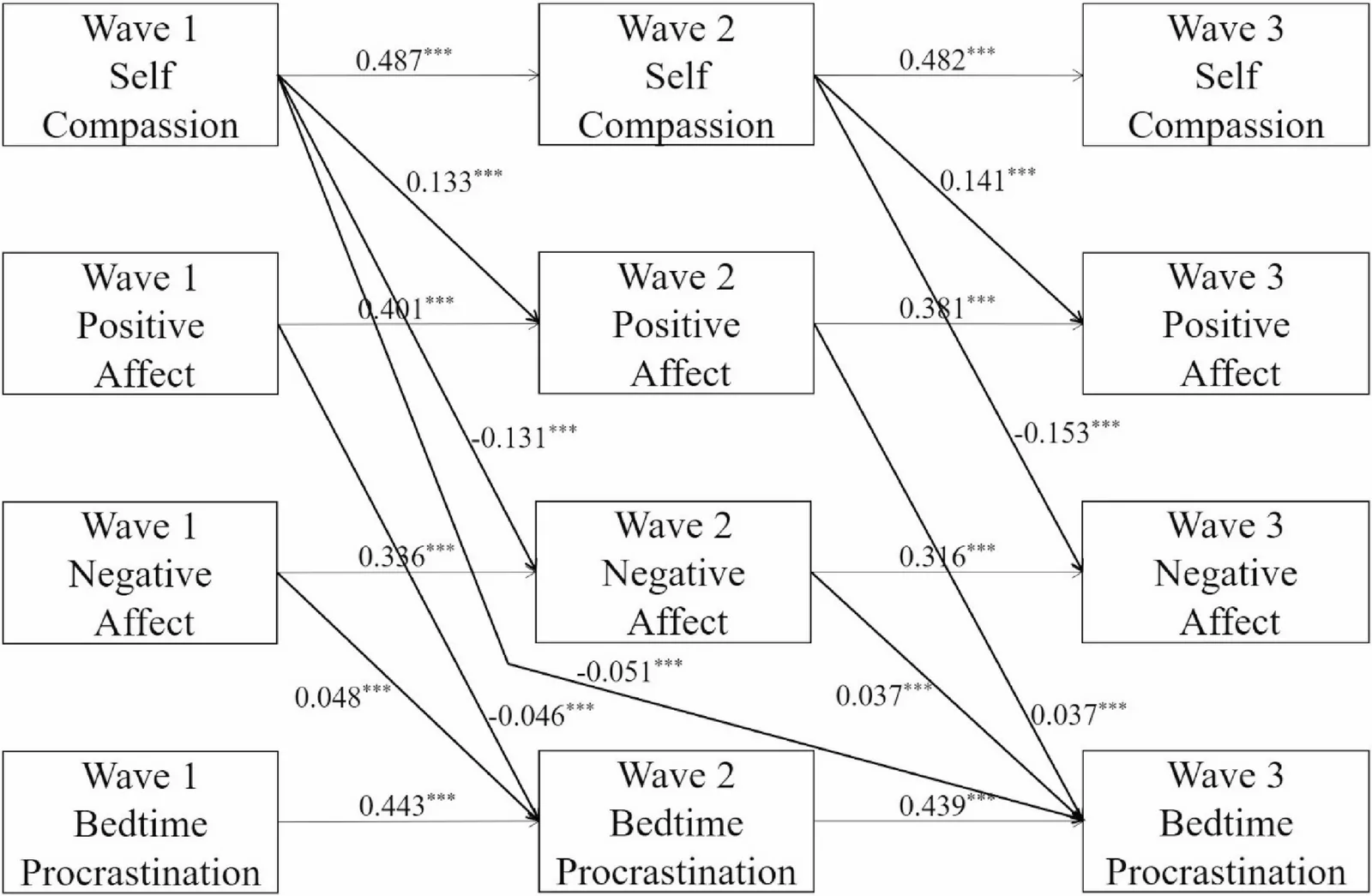
Cross-lagged panel model with mediation. Note: For clarity, coefficients for the direct paths along the hypothesized indirect pathway, and the direct path from Wave 1 self compassion to Wave 3 bedtime procrastination are in bold. Standardized results are listed. ^***^p < 0.001

The CLPM model showed evidence of good fit with the data: *χ*^*2*^ [31] = 1913.942 (*p* < 0.001), RMSEA = 0.078 (90% CI 0.075–0.082), SRMR = 0.071, CFI = 0.96, TLI = 0.92. Results indicated the relationships among self-compassion, positive affect, negative affect, and bedtime procrastination over three time points (Fig. 1). Significant autoregressive effects indicated stability within individuals over time for all three constructs. Cross-lagged effects showed that the indirect links from self-compassion to bedtime procrastination via positive affect (*β* = -0.007, *p* < 0.001) and negative affect (*β* = -0.005, *p* < 0.001) were significant. Results based on the full sample using Full Information Maximum Likelihood (FIML) were highly consistent with those obtained from the complete-case sample, supporting the robustness of the findings (see Table S2 in the Supplementary Materials).

## Discussion

The present study is the first to employ a three-wave longitudinal design to examine the mediating roles of both positive and negative affect in the association between self-compassion and bedtime procrastination. Consistent with our hypotheses and extending prior cross-sectional findings [16, 23], the results demonstrated that self-compassion predicted subsequent reductions in bedtime procrastination, and this relationship was significantly mediated by both increased positive affect and decreased negative affect. These findings confirm the central tenet of the TASRR model [17], which posits that adaptive emotional states act as self-regulatory resources that facilitate health-promoting behaviors and inhibit health-compromising behaviors.

Our findings provide new empirical evidence that both positive and negative affect function as significant mediators in the relationship between self-compassion and bedtime procrastination. While prior cross-sectional studies have consistently identified negative affect as a mediating factor, they did not observe a significant mediating role for positive affect [16, 23]. These studies attributed the asymmetry to the possibility that self-compassion measures are more strongly associated with negative emotional states than with positive ones. However, despite the absence of a mediating effect, their results still indicated that individuals with higher self-compassion reported both higher levels of positive affect and lower levels of negative affect. In contrast, the present study employed a three-wave longitudinal design with cross-lagged panel modeling, which allowed for the examination of temporal precedence and dynamic change. This methodological approach offers a more robust test of mediation by capturing fluctuations and directional relationships over time. Our findings provide longitudinal evidence supporting the mediating roles of both positive and negative affect in the association between self-compassion and bedtime procrastination. These results underscore the importance of considering both affective pathways and highlight the added explanatory power of longitudinal data in uncovering mechanisms that may be obscured in cross-sectional designs [34]. However, these associations should not be interpreted as strictly unidirectional, as alternative temporal pathways and reciprocal relationships may also exist. Indeed, alternative model testing suggested that reversed mediation pathways were also plausible, although with slightly weaker model fit, indicating the potential existence of a dynamic and mutually reinforcing relationship between these constructs over time.

This dual affective pathway aligns with emotion regulation theories that underscore the role of both upregulating positive and downregulating negative emotions in adaptive functioning [35]. From this perspective, individuals with higher levels of self-compassion are more likely to engage in adaptive regulatory strategies such as cognitive reappraisal, acceptance, and mindfulness. These strategies enable them to interpret stressors and setbacks in ways that alleviate emotional distress and promote psychological resilience [36, 37]. By promoting a stable, internal source of affective regulation, self-compassion may reduce individuals’ reliance on short-term, externally driven strategies like bedtime procrastination, which offer immediate mood repair at the cost of long-term health [15]. Over time, this capacity for sustained emotional regulation may help break the cycle in which individuals habitually delay bedtime in response to daily stressors, instead enabling them to meet their sleep needs in a more intentional and health-supportive manner.

This study also extends the understanding of bedtime procrastination by situating it within an emotion regulation framework. Bedtime procrastination has been conceptualized as a self-regulation failure wherein individuals sacrifice long-term goals (i.e., adequate sleep) for short-term emotional comfort [16]. Importantly, our results suggest that this cycle can be interrupted through the cultivation of self-compassion and its associated emotional benefits. In addition, recent research indicates that bedtime procrastination may stem from two distinct but potentially overlapping pathways: a reward-driven pathway characterized by difficulties disengaging from enjoyable pre-sleep activities, and an avoidance-driven pathway motivated by attempts to escape pre-sleep anxiety and negative thoughts [14]. These two motivations reflect different facets of emotional dysregulation—either through heightened approach sensitivity to rewarding stimuli or through maladaptive avoidance of negative affect. Self-compassion may play a unique regulatory role in both pathways: by promoting greater mindful awareness and acceptance, it may reduce the urge to overindulge in stimulating activities, and by buffering against anxiety and self-critical ruminations, it may decrease the perceived need to delay sleep as a form of emotional escape [36, 38]. Future studies would benefit from explicitly disentangling these mechanisms, as interventions targeting reward sensitivity (e.g., behavioral activation) versus those addressing avoidance (e.g., anxiety reduction or exposure-based approaches) may require different therapeutic strategies. Integrating self-compassion into such interventions may offer a flexible and broadly applicable means to address both pathways simultaneously.

To note, although the indirect paths through affect were statistically significant, the direct effect of self‑compassion on bedtime procrastination remained robust. This suggests that additional mechanisms beyond affect regulation may account for why self‑compassionate individuals are less likely to delay bedtime. For instance, self-compassion has been linked to future-oriented coping [39], which may independently reduce procrastinatory behavior [40]. As such, even in the absence of immediate emotional distress, self-compassion may exert a direct influence on behavior by fostering self-regulatory patterns that are organized around future well-being rather than short-term relief. Another potential mechanism is the activation of a self‑soothing physiological system. Self‑compassion can activate a sense of safety and relaxation, counterbalancing threat‑based arousal systems that interfere with sleep onset [41]. By promoting physiologic calm, self‑compassion may directly support sleep readiness. Future studies should investigate other potential mediators, such as future-oriented coping or pre-sleep arousal, to more fully elucidate this relationship. In addition, although the indirect effects via positive and negative affect were statistically significant, the magnitude of these effects was relatively small (*β* = −0.007 and *β* = −0.005). This indicates that the mediating role of affect, while reliable across waves, may reflect subtle and gradual processes rather than strong or immediate causal influences on bedtime procrastination. Such small effect sizes are not uncommon in longitudinal studies of complex behavioral outcomes, which are typically shaped by multiple interacting psychological, contextual, and environmental factors. From an applied perspective, these findings suggest that interventions targeting self-compassion and affect regulation may produce modest but potentially cumulative benefits over time rather than large short-term behavioral changes. Importantly, even small effects may still hold practical significance when scaled to population-level or digital mental health interventions, where repeated micro-level changes can accumulate into meaningful behavioral improvements.

Despite its strengths, including a large sample size and longitudinal design, several limitations warrant mention. First, affect was measured at discrete intervals rather than in real-time or close to the bedtime procrastination episodes. While trait-level affect has been shown to be relatively stable and predictive of behavior [42], experience sampling or daily diary methods could provide more granular insights into how fluctuations in affect relate to bedtime procrastination on a day-to-day basis. Second, while the sample was large and diverse in terms of academic disciplines, participants were drawn from a single university in China, potentially limiting the generalizability of the findings. Future research should replicate these findings in other cultural contexts and with different age groups, such as adolescents or working adults, who may experience different pressures around bedtime behavior. Third, although the longitudinal design allows for stronger inferences regarding temporal ordering, the observational nature of the data precludes definitive conclusions about causality. Experimental or intervention-based studies that actively manipulate self-compassion or affective states would provide more rigorous tests of the proposed mechanisms. Fourth, Although bedtime procrastination has been conceptualized as a self-regulation failure and prior research has identified self-control as an important correlate of this behavior, the present study concentrated only on emotional pathways in order to test the specific affect-based mechanisms proposed by the TASRR model. Future research would benefit from integrating both self-control and affective resources to provide a more comprehensive account of the self-regulatory processes underlying bedtime procrastination. Finally, the longitudinal associations were examined using traditional cross-lagged panel modeling, which does not distinguish between within-person and between-person effects. More advanced approaches such as Random Intercept Cross-Lagged Panel Model (RI-CLPM) were not implemented in the final analyses due to model complexity constraints. Therefore, caution is warranted when interpreting the present findings as reflecting within-individual temporal dynamics. Future research adopting RI-CLPM or related decomposition approaches is needed to provide a more fine-grained understanding of these associations.

## Conclusions

The present study provides the first longitudinal evidence that both positive and negative effects mediate the relationship between self-compassion and bedtime procrastination. These findings highlight the central role of emotional processes in understanding and potentially modifying bedtime procrastination. Cultivating self-compassion may be a promising intervention target for promoting healthier sleep behaviors by enhancing emotional regulation capacities. Future experimental work is needed to further unpack these mechanisms and explore the efficacy of self-compassion-based interventions in reducing bedtime procrastination.

## Supplementary Information

Supplementary Material 1.

## Data Availability

The data and code used in this study is available from the corresponding author on reasonable request.

## Abbreviations

TASRR: Temporal Affective Self-Regulation Resource
SCS-SF: Self-Compassion Scale-Short Form
PANAS: Positive and Negative Affect Schedule
BPS: Bedtime Procrastination Scale
CLPM: Cross-lagged Panel Model
CFI: Comparative Fit Index
TLI: Tucker-Lewis Index
RMSEA: Root Mean Square Error of Approximation
SRMR: Standardized Root Mean Square Residual
