# Supplementary material for: Self-compassion predicts reduced bedtime procrastination through affect pathway: a three-wave longitudinal study
Source: BMC Psychol. 2026 Jul 31;14:1552. doi: 10.1186/s40359-026-05267-x (PMC13712021; doi:10.1186/s40359-026-05267-x)
Supplement: Supplementary file 1 [file 40359_2026_5267_MOESM1_ESM.docx]

Table S1. Attrition Analysis

| Variable | Retained (*n* = 7174)  *M* ± *SD* | Dropout (*n* = 3965)  *M* ± *SD* | *t*/*χ²* | *p* |
| --- | --- | --- | --- | --- |
| Age | 19.11 ± 1.30 | 19.16 ± 1.32 | 1.80 | 0.07 |
| Female (%) | 62.82 | 61.03 | 3.48 | 0.06 |
| Self-compassion at T0 | 44.67 ± 7.24 | 44.71 ± 7.36 | 0.28 | 0.78 |
| Positive affect at T0 | 32.37 ± 7.32 | 32.23 ± 7.44 | -1.01 | 0.31 |
| Negative affect at T0 | 19.77 ± 6.05 | 20.00 ± 6.36 | 1.83 | 0.07 |
| Bedtime procrastination at T0 | 26.21 ± 7.51 | 26.33 ± 7.60 | 0.84 | 0.40 |

| **Path** | **Complete Cases**  **(*n* = 7174)** | **Full Information Maximum Likelihood (*n* = 11139)** |
| --- | --- | --- |
| SCS→PA | 0.133*** | 0.149*** |
| PA→BPS | -0.054*** | -0.055*** |
| Indirect (SCS→PA→ BPS) | -0.007*** | -0.008*** |
| SCS→NA | -0.131*** | -0.145*** |
| NA→BPS | 0.041*** | 0.042*** |
| Indirect (SCS→NA→ BPS) | -0.005*** | -0.006*** |
| Direct | -0.093*** | -0.092*** |

Table S2. Comparison of path coefficients between complete-case analysis and full-information maximum likelihood (FIML) estimation

*Note.* SCS = self-compassion; PA = positive affect; NA = negative affect; BPS = bedtime procrastination. Standardized path coefficients are reported. *** *p* < .001.
